# A measure for the promotion of mountain ecological villages in South Korea: focus on the national mountain ecological village investigation of 2014

**DOI:** 10.1186/s40064-016-2206-5

**Published:** 2016-05-11

**Authors:** Soo Im Choi, Hag Mo Kang, Hyun Kim, Chang Heon Lee, Chong Kyu Lee

**Affiliations:** Department of Forest Resources, Sunchon National University, Suncheon, 57922 South Korea; Department of Forest Environmental Science, Chonbuk National University, Jeonju, 54896 South Korea; Jeollabuk-do Forest Environment Research Institute, Jinan, 55454 South Korea; Department of Forest Resources, Gyeongnam National University of Science and Technology, Jinju, 52725 South Korea

**Keywords:** Farm/rural-returning population, Income source development, Mountain ecological village, Promotion measure

## Abstract

**Background:**

Although South Korean mountain villages occupy 44 and 55 % of land and forest areas, respectively, these villages account for only 3 % of the national population and they suffer from a declining workforce owing to aging, wage inflation, and low forestry productivity. As a result, the South Korean government implemented a mountain ecological village development project from 1995 to 2013 in 312 of the 4972 mountain villages and investigated project performance in 2014. The present study establishes a measure for the promotion of mountain ecological villages by comparing the situation before and after the project.

**Results:**

The analysis found a threefold increase in the inflow of farm/rural-returning and multicultural households compared with before the project, while the average income per farm, local product sales, and experience tourism revenue also grew remarkably every year. In addition, households utilizing forest resources increased by about 30 %, but 45.8 % of the 312 villages had no long-term plan for village development and villagers experienced low satisfaction with job creation and village income.

**Conclusions:**

A systematic revision of agroforestry production and forest administration is needed to define the characteristics of farm/rural-returning populations clearly, reorganize urban–rural exchange and experience programs, and reinforce tangible/intangible cultural assets and religious traditions.

## Background

The South Korean land area is 10.019 million ha, of which forest areas account for 6.422 million ha, or 64 %. South Korea has a temperate climate; the average annual temperature in its mountain villages is 12.5 °C, annual precipitation is 1113 mm, annual snowfall days are 33 days, and the average altitude of South Korean mountain villages is 248 m above sea level (range of 137–484 m) (Korea Forest Service 2014c). In addition, although South Korean mountain villages occupy 44 and 55 % of the national land area and forest area, respectively, their populations account for only about 3 % of the national population. Moreover, the economies of mountain villages have been struggling because of such factors as declining workforce due to aging population, wage inflation, and low forestry productivity. Nevertheless, mountain villages play important roles, including ensuring stable agroforestry production, balanced national land development, forest administration, and the continuation of forests’ cultural and traditional heritages. Therefore, the South Korean government carried out a development project for 312 of the 4972 mountain villages in South Korea from 1995 to 2013.

The project has improved income and the residential environment, increased the population, and established a system for the stable promotion of the mountain village development project. However, some issues remain, including insufficient long-term development plans, unsatisfactory development and branding of special mountain village products, problems with the operation and management of facilities after project completion, and insufficient follow-up support. Furthermore, several suggestions have been proposed for the promotion of mountain villages, including the expansion of the fund for the promotion of mountain villages, active participation by local residents, development of customized projects according to the characteristics of mountain villages, and expansion of forest product utilization to raise income (Kang 2007). Meanwhile, mountain ecological villages have laid the foundations for promoting various experience programs and offering them to urban residents to raise the income levels of village residents. For instance, to expand urban–rural exchange programs, villages have been identifying and developing not only tangible assets, such as traditional forests, guardian trees, waterwheels, traditional farming tools, waterfalls, wetlands, temples, and cultural properties, but also intangible assets, such as the history and folklore of villages, religious rituals, traditional Korean music, folk music, and traditional festivals.

Several studies of mountain villages were performed in the 1990s (Lee 1986; Chang and Choi 1989; Kim and Shon 1994, 1995; Choi et al. 1998; Kang et al. 1998, 1999) and 2000s (Shon and Chang 2000; Han 2001; Kwak and Kim 2002; Eom 2004; Kim et al. 2004; Moon 2004; Yoo et al. 2004; An et al. 2005; Jeon and Chung 2007; Jeon et al. 2007; Chang 2008; Han and Seol 2008; Lee et al. 2008; Yun and Kim 2008; Seo et al. 2009). Since 2010, however, the findings of such studies have shown a remarkable change in the perspectives of mountain villages over time (Jeong et al. 2010; Kim et al. 2010; Seo and Lee 2010; Seo et al. 2011; Kwak and Seo 2012; Kim and Seo 2013; Kim et al. 2013a, 2013b; Roh et al. 2013; Chang et al. 2014; Kim and Seo 2014a, 2014b; Min and Kim 2014; Seo et al. 2015). In particular, the populations of mountain villages have decreased continuously, and average income per household is now about half the national average. In addition, only 52,000 of the nation’s 682,000 mountain village households utilize forest resources, corresponding to only 7.6 % of all households. Moreover, obstacles to mountain village revitalization include deficiencies of customized projects according to village characteristics and lack of a main mission for each project (Korea Forest Research Institute 2003). Therefore, the present study aims to establish a measure for the promotion of mountain ecological villages based on the results of a 2014 investigation (Korea Forest Service 2014b) performed on mountain ecological village development project. This investigation included 312 mountain villages from 1995 to 2013 and examined the effective promotion and fundamental planning of the policy for the promotion of mountain villages in South Korea.

## Methods

The status of mountain villages and mountain ecological village development project in South Korea were investigated using related laws, previous studies, statistical data from related agencies, and online sources. The mountain ecological village investigation examined the 312 mountain ecological villages that finalized development from 1995 to 2013. The investigation was led by professors of forest policy from seven universities in order to increase the objectivity and reliability of the investigation (Korea Forest Service 2014b).

The major components of the investigation:

- Population and land use (households and population, population by age, village migrants, and land use);
- Infrastructure and income (housing infrastructure supply ratio for villages, revenue, including average income, and households utilization of forest resources);
- Promotion of the mountain ecological village development project (satisfaction with the development project, follow-up management and support for the development project, project performance factors, and factors for continuous operation and management); and
- Operation and planning of mountain ecological villages (operation manager, website operation, long-term plan for village developments, residents’ empowerment, development potential using forest resources, village income satisfaction, demand for revenue-making projects using forests, and desired support items).

Then, the results before and after the mountain ecological village development project at the end of 2013 were comparatively analyzed. The survey results were statistically analyzed using PASW 18.0 (SPSS Inc., Chicago, IL, US), and the Chi square independence test, independent samples t test, one-way analysis of variance (ANOVA), and Chi square goodness of fit test were conducted at 95 % confidence intervals.

### Background of the mountain ecological village development project

The South Korean government defines mountain villages as villages located in remote areas in mountains. They are characterized by high ratios of forestland occupation and low income levels, have a weak industrial foundation because of their isolation from socioeconomic and cultural facilities, have low standards of living, and are located in depopulated areas. However, the classification standard of mountain villages changed following the enactment of the Framework Act on Forestry in 2001. This Act specified that the forest area ratio of the Eup/Myeon administrative district area should be 70 % or higher, that the population density for Eup/Myeon should be below the national average, and that the agricultural area ratio of the administrative district for Eup/Myeon should be below the national average (Korea Forest Service 2015).

When the mountain ecological village development project was carried out in 1995, mountain villages in South Korea occupied 46 % of the national land area (46,181 km^2^), 58 % of the forest area (3.746 million ha), and 27 % of the agricultural area (548,000 ha), whereas its population accounted for as little as 4.6 % of the whole population (1.94 million individuals). Therefore, the Korean government initiated mountain ecological development project by selecting one village in 1995 as the test case for the purposes of balanced national land development, efficient use and management of forests, and the development of backward mountain villages. The project aims to provide clean food products to Koreans and raise rural household income to the extent that the collection of forest products causes no harm to the forest ecosystem. The government defines a mountain ecological village as follows: “It is located in a remote mountain area or has abundant forest resources and has a good mix of well-preserved ecological environment and recreational culture” (Korea Forest Service 2010). Therefore, any mountain villages willing to participate in the project should fall under the definition.

Nevertheless, mountain ecological village development project experienced some operational difficulties because of budget constraints. However, the Framework Act on Forestry of 2001 and the Forestry and Mountain Village Promotion Act (Revised) of 2001 prepared detailed provisions on the promotion of mountain villages, enabling the development project to operate more systematically and stably. Meanwhile, the government performed its first national mountain village investigation in 2003 and the second investigation in 2014 in order to secure basic data for the effective promotion and planning of village promotion policy (Korea Forest Service 2015).

The national mountain village investigation is required to be performed every 10 years. The first national investigation was conducted with 4972 of the country’s 15,277 mountain villages, while the second was conducted with 466 Eup/Myeon mountain villages (109 Si/Gun) based on Articles 2 and 3 of the Framework Act on Forestry. The content of the first national mountain village investigation in 2003 included the distribution and utilization of forests and farmland, village distributions and trends of population variation, resources of green tourism and ecotourism, infrastructure for agroforestry production, medical facilities, amenities, and items required for other basic plans. The second national mountain village investigation in 2014 added such content as information on the income of mountain villages, workforce (farm/rural-returning and multicultural migrants), and urban–rural exchanges (Korea Forest Service 2014a, 2014c, 2014d).

### Status of mountain ecological village development project

The mountain ecological village development project started in 1995 by targeting 4972 mountain villages of 119 Si/Gun in South Korea. Four types were promoted, considering local characteristics, as follows: recreation-linked, forest income, mixed agriculture and forestry, and comprehensive development types. In total, 310.1 million USD (about 1.3 million USD per village on average) was invested in 240 villages (17,614 households) as promotion funds from 1995 to 2010. Of these funds, project funds (70 % government funds and 30 % local funds) were used for residential environment improvements (106.3 million USD, 34 %), production base developments (103.2 million USD, 33 %), eco-tourism facilities (75.9 million USD, 25 %), and miscellaneous projects (24.7 million USD, 8 %). The provision of project funds depended on the number of village households, and about 413.9 million USD was invested in 312 villages as development project funds by 2013 (Seo et al. 2013).

Residential environment improvements focused on roads and pavements in villages, river maintenance, streetlights, wastewater treatment facilities, street trees, and village halls. Production base developments focused on short-term income increases, including via cold storage facilities, income production projects (mushrooms, wild vegetables, etc.), and storage facilities for collection of forest products. Eco-tourism facilities included accommodation, experience facilities, forest parks, and hiking trails. Finally, miscellaneous projects included house improvements, new construction of houses, residents’ education, nurturing of village leaders, website construction, and village advertising (Kang 2004).

The Special Act on Balanced National Development was revised to resolve problems, including negligence in the operation and management of facilities and overlapping with other development projects from different areas. In addition, it aimed to refresh connections with local governments so that the mountain ecological village development project of the Korea Forest Service was operated broadly under a regional development framework at the village level (Korea Forest Research Institute 2003; Korea Forest Service 2014a; Seo and Kim 2014, 2015). Meanwhile, the South Korean government examined the 312 mountain ecological villages for the first time in 2014 to understand the performance of the mountain ecological village development project.

## Results and discussion

### Population and land use

Before the project, there were 21,039 households in the 312 villages that successfully implemented the mountain ecological village development project in South Korea, whereas this increased to 23,883 in 2013, an increase of 13.5 %. However, the additional households were mostly non-farm and non-resident households rather than farms. Thus, the changes in the number of households for each type of residence were statistically significant (p < 0.001). The population increased slightly after the project compared with before (0.5 %); the proportion of women increased by 1.2 %, while that of men decreased by 0.2 %. However, there was no statistically significant difference between the population changes and the aim of the project (p > 0.05; Table 1).

**Table 1:** Households and population

Division	Households (household)						Population by gender (person)			
	Residence			Non-residence	Total	*χ* ^2d^	Men	Women	Total	*χ* ^2^
	Farm^a^	Non-farm^b^	Subtotal							
Before project^c^	16,734 (79.5)	3922 (18.6)	20,656 (98.2)	383 (1.8)	21,039 (100.0)	548.665***	24,665 (49.5)	25,210 (50.5)	49,875 (100.0)	1.066
After project	16,864 (70.6)	5993 (25.1)	22,857 (95.7)	1026 (4.3)	23,883 (100.0)		24,626 (49.1)	25,501 (50.9)	50,127 (100.0)	
Variation	130 (0.8)	2071 (52.8)	2201 (10.7)	643 (167.9)	2844 (13.5)		−39 (−0.2)	291 (1.2)	252 (0.5)	

Values in parentheses indicate percentage compositions

^a^Households with full-time jobs in agriculture and forestry

^b^Households with full-time jobs other than in agriculture and forestry

^c^The number of mountain ecological villages that completed the project was 25 between 1995 and 2000, 215 between 2001 and 2010, and 72 between 2011 and 2013; “Before project” means the year before the development project started

^d^Chi square independence test, * p < 0.05, ** p < 0.01, and *** p < 0.001

In terms of the population change by age, the age group of 70 years or older accounted for the highest ratio (20 %) before the project, while the 60s or older age group occupied more than one-third of the total population (38.6 %). Similarly, the 70s or older age group accounted for the highest ratio (23 %) after the project, while the 60s or older age group occupied 41.1 % of the total population, an increase of 2.5 % compared with the age ratio before the project. Thus, the population changes in each age group after the project were statistically significant (p < 0.001). The populations of mountain ecological villages after the project decreased in all age groups except the 50s and 70s or older groups, which reflects the common characteristics of modern South Korean society, namely, the aging of rural and mountain village populations and the outflow of younger populations to urban areas (Table 2).

**Table 2:** Population by age

Division	Population by age (person)								
	10s	20s	30s	40s	50s	60s	≥70s	Total	*χ* ^2a^
Before project	5608 (11.2)	4192 (8.4)	4758 (9.5)	7073 (14.2)	8990 (18.0)	9299 (18.6)	9955 (20.0)	49,875 (100.0)	267.827***
After project	5244 (10.5)	3646 (7.3)	4157 (8.3)	6573 (13.1)	9903 (19.8)	9092 (18.1)	11,512 (23.0)	50,127 (100.0)	
Variation	−364 (−6.5)	−546 (−13.0)	−601 (−12.6)	−500 (−7.1)	913 (10.2)	−207 (−2.2)	1557 (15.6)	252 (0.5)	

Values in parentheses indicate percentage compositions

^a^Chi square independence test, * p < 0.05, ** p < 0.01, and *** p < 0.001

In terms of village migrants, farm/rural-returning households increased by 943 households (187.1 %) and 2028 individuals (199.4 %) compared with those before the project. In addition, multicultural families increased by 135 households (157 %) and 332 individuals (212.8 %) compared with those before the project (Table 3). Farm/rural-returning households and multicultural families were analyzed as a unique class that countervailed the population outflow rate of mountain ecological villages.

**Table 3:** Status of village migrants

Division	Farm/rural-returning households^a^				Multicultural families^b^			
	Number of households (household)	Men (person)	Women (person)	Total (person)	Number of households (household)	Men (person)	Women (person)	Total (person)
Before project	504	523 (51.4)	494 (48.6)	1017 (100.0)	86	59 (37.8)	97 (62.2)	156 (100.0)
After project	1447	1506 (49.5)	1539 (50.5)	3045 (100.0)	221	206 (42.2)	282 (57.8)	488 (100.0)
Variation	943 (187.1)	983 (188.0)	1045 (211.5)	2028 (199.4)	135 (157.0)	147 (249.2)	185 (190.7)	332 (212.8)

Values in parentheses indicate percentage compositions

^a^People who used to have other jobs and migrated to mountain villages for farming or settlement

^b^Family composed of members with different nationalities, races, or cultures

Forests commonly accounted for more than 79 % of land before and after the mountain ecological village development project. However, 446 ha of forest and 1048 ha of farmland were reduced after the project. These reduced areas were found to have been transformed into rice paddies and other uses (sites, roads, and land for facilities) (Table 4).

**Table 4:** Land use

Division	Farm land (ha)			Forest (ha)	Other (ha)^a^	Total (ha)
	Field	Rice paddy	Subtotal			
Before project	17,127 (3.1)	24,022 (4.3)	41,149 (7.4)	442,801 (79.3)	74,581 (13.4)	558,531 (100.0)
After project	16,079 (2.9)	24,558 (4.4)	40,637 (7.3)	442,355 (79.2)	75,655 (13.5)	558,647 (100.0)
Variation	−1048 (−6.1)	536 (2.2)	−512 (−1.2)	−446 (−0.1)	1074 (1.4)	116 (0.0)

Values in parentheses indicate percentage compositions

^a^Land categorized as other than farmland and forest

### Infrastructure and income

Although water supply and sewers (i.e., housing infrastructure) increased by 26.7 and 23.5 %, respectively, compared with before the project, sewer supply was just over 50 %, implying that sewers needed to be supplied additionally to improve the life quality of mountain ecological village residents. By contrast, flushable toilets increased by about 30 %. The changes in the ratios of the three kinds of housing infrastructure supply after the project were statistically significant (p < 0.001; Table 5). Better toilet and sewage treatment were provided to avoid direct inflow of household sewage to rivers and streams, thereby playing an important role in preventing water and environmental pollution in mountain ecological villages.

**Table 5:** Housing infrastructure supply ratio to villages

Division	Water supply	t^a^	Sewer	t	Flushable toilet	t
Before project (%)	37.8	−3.821^***^	26.9	−3.737***	44.1	−3.930***
After project (%)	64.5		50.3		73.6	
Variation (%p)	26.7		23.5		29.5	

%p (percentage point) is the unit for the arithmetic difference of two percentages

^a^Independent samples t test, * p < 0.05, ** p < 0.01, and *** p < 0.001

Average income per household and per farm increased by 23.2 and 27.8 %, respectively, at the end of 2013 compared with before the project (20,307 USD and 20,534 USD). However, there was no statistically significant difference between the changes in average income per household and average income per farm and the project (p > 0.05). On the other hand, the change in average income per capita after the project was statistically significant (p < 0.05). Meanwhile, the average farm income accounted for 97.5 % of the average income per household in all mountain ecological village households before the project, and this increased more than the average income per household after the project (Table 6). Hence, the mountain ecological village development project can be considered to have contributed to the increase in farm income.

**Table 6:** Average income

Division	Average income per household^a^ (A) (USD)	t^d^	Average income per person^b^ (USD)	t	Average income per farm^c^ (B) (USD)	t	B/A
Before project	16,488	−1.706	7126	−2.266*	16,071	−1.431	97.5
After project	20,307		9959		20,534		101.1
Variation	3819 (23.2)		283 (39.8)		4463 (27.8)		116.9

Values in parentheses indicate percentage compositions

^a^Income by the calculation of total village income divided by the number of households

^b^Income by the calculation of total village income divided by the number of residents

^c^Average income of households that have agriculture as a full-time job

^d^Independent samples t test, * p < 0.05, ** p < 0.01, and *** p < 0.001

Total revenue from local product sales and experience tourism in mountain ecological villages were 54,626 USD and 47,412 USD, respectively, for 2013–2015. Total revenue increased by 133.1 % in 2012 and 185.5 % in 2013 for local products and 145.3 % in 2012 and 297.7 % in 2013 for experience tourism. However, although net revenue for these 3 years were 50.9 % of total revenue for local products and 48.7 % for experience tourism, net revenue rates decreased in 2013, which might have been because of the increases in direct/indirect production costs and labor costs (Table 7). Furthermore, in general, experience programs and facilities are needed for experience tourism. Examples of such programs include participative village festivals, with such activities as harvesting and tasting of agricultural and forest products, barbecues, woodcraft, rafting, lodging at local houses, camping in villages, observing fish and insects, stargazing, sledding, and listening to the history and legends of villages. Depending on the characteristics of each village, the facilities include accommodation, experience program centers, campgrounds, barbeque areas, snow sledding fields, forest bathing spots, walking trails, and hiking trails.

**Table 7:** Revenue of mountain ecological villages

Division	2011 (USD)	2012 (USD)	2013 (USD)	Total (USD)
Local products				
Total revenue^a^	13,050 (100.0)	17,375 (133.1)	24,202 (185.5)	54,626
Net revenue^b^	7691 (100.0)	10,625 (138.1)	9485 (123.3)	27,801
Experience tourism				
Total revenue^c^	8731 (100.0)	12,688 (145.3)	25,993 (297.7)	47,412
Net revenue^d^	6937 (100.0)	9654 (139.2)	6519 (94.0)	23,110

Values in parentheses indicate percentage compositions

^a^Total monetary income that producers obtained from local product sales or experience tourism

^b^Amounts of money, excluding direct production costs invested for crop production (expenses for seeds, fertilizers, agricultural chemicals, and other expenses) and indirect production costs (rent and interest) from total revenue

^c^Total monetary income from experience tourism, including accommodation, food, and programs

^d^Amounts of money, excluding material costs, labor costs, and operation costs, from total revenue through experience tourism

The number of households utilizing forest resources was 8432 after the project, corresponding to a 28.7 % increase from the 6554 households before the project. The number of households utilizing forest resources per village in all 312 mountain ecological villages increased from 21 before the project to 27 households after. Forest resources utilized after the project were wild greens (32.3 %), fruit trees (25.2 %), and forest mushrooms (16.6 %), while forest resources with the highest rates of increase after the project compared with those before were landscaping trees (303.5 %), medicinal plants (59.7 %), and wood-cultivated ginseng (57 %). On the contrary, wood materials decreased by 50 %, and wild birds and beasts showed no change. The changes in the number of households utilizing forest resources after the project were statistically significant (p < 0.001; Table 8). Furthermore, although harvesting wild greens and medicinal plants has been one of the sources of household income of mountain villages for a long time, the goals of the project are to provide clean food products to Koreans and to increase the household income of mountain ecological villages without interrupting the forest ecosystem.

**Table 8:** Households utilizing forest resources

Division	Wild greens (house-hold)^a^	Fruit trees (house-hold)^b^	Forest mush-rooms (house-hold)^c^	Sap (house-hold)^d^	Medicinal plants (house-hold)^e^	Wood-cultivated ginseng (house-hold)	Land-scaping trees (house-hold)	Timber (house-hold)^f^	Wild birds and beasts (household)^g^	Total (house-hold)	*χ* ^2h^
Before project	2215 (33.8)	1617 (24.7)	1273 (19.4)	578 (8.8)	457 (7.0)	291 (4.4)	57 (0.9)	34 (0.5)	32 (0.5)	6554 (100.0)	119.019***
After project	2723 (32.3)	2129 (25.2)	1397 (16.6)	717 (8.5)	730 (8.7)	457 (5.4)	230 (2.7)	17 (0.2)	32 (0.4)	8432 (100.0)	
Variation	508 (22.9)	512 (31.7)	124 (9.7)	139 (24.0)	273 (59.7)	166 (57.0)	173 (303.5)	−17 (−50.0)	0 (0.0)	1878 (28.7)	

Values in parentheses indicate percentage compositions

^a^Wild greens include deodeok, bracken, balloon flower, and chwinamul

^b^Fruit trees include chestnut, persimmon, walnut, pine nut, and jujube

^c^Forest mushrooms include pyogo mushroom, pine mushroom, and wood ear

^d^Sap includes *Acer mono* and birch

^e^Medicinal plants include barrenwort, peony, the root bark of various araliaceous shrubs, and a Chinese matrimony vine

^f^Wood materials include timber production (thinned wood/main cutting wood)

^g^Wild birds and beasts include pheasants, wild boar, elk, and deer

^h^Chi square independence test, * p < 0.05, ** p < 0.01, and *** p < 0.001

### Mountain ecological village development project

Satisfaction with the sub-projects of the mountain ecological village development project was investigated with village representatives and residents using a five-point scale. Residential environment improvement projects scored 3.5 points corresponding to the highest satisfaction, followed by income-based development projects with 3.3 points and income source development and welfare facility projects with 3.1 points each. House improvement projects garnered the lowest satisfaction among sub-projects because residents were found to be satisfied with the surrounding residential environment improvements and income-related projects. These differences were statistically significant (p < 0.01; Table 9).

**Table 9:** Satisfaction with the development projects

Division	Satisfaction (5-point scale)	F^c^
Residential environment improvement	3.5 ± 0.8^a^	13.000***
Income-based development	3.3 ± 0.9^a^	
Income source development	3.1 ± 0.9^b^	
Welfare facility	3.1 ± 0.9^b^	
House improvement	3.0 ± 0.8^b^	

^a, b^Groups by Duncan’s post hoc analysis

^c^One-way ANOVA, * p < 0.05, ** p < 0.01, and *** p < 0.001

With regard to management, including facility management or business guidance after the mountain ecological village development project, administrative agencies managed 74.7 % of the 312 villages, while 82.7 % of the villages managed themselves. In addition, 76.6 % of all villages operated facilities based on village funds. Although residents prepared their own measures for the management and operation of the project, the follow-up support ratio from the government for forests and environmental improvements was as low as 29.1 %. Hence, government-led follow-up support was considered to be required (Table 10).

**Table 10:** Follow-up management of and support for the development project

Division	Present (village)	Absent (village)	Total (village)
Management by administrative agencies	233 (74.7)	79 (25.3)	312 (100.0)
Management and project promotion by village itself	258 (82.7)	54 (17.3)	312 (100.0)
Development and operation of own village funds for facility management	239 (76.6)	73 (23.4)	312 (100.0)
Follow-up support by the government for forests and environmental improvements	122 (39.1)	190 (60.9)	312 (100.0)

Values in parentheses indicate percentage compositions

The most effective factor in project performance was understanding and cooperation by residents (40.1 %), followed by shared income creation and government support, accounting for 20.5 % each. The differences in the factors were statistically significant (p < 0.001). On the effect of the mountain ecological village development project on villages, the maximization of the forest environmental characteristics of villages (33 %) was the highest, followed by the maximization of the utilization of forest resources (24.7 %), achievement of competitiveness (22.1 %), and increased migration of urban residents (17.6 %). The differences in the effects were statistically significant (p < 0.001). For the sustainable operation and management of mountain ecological villages, 43.3 % answered that additional support from the government was required, followed by continuous resident education and advertisement (16 %) and the inflow of younger people through a farm/rural-returning induction policy (10.3 %). The differences in the prerequisites were statistically significant (p < 0.001; Table 11).

**Table 11:** Project performance factors and factors for continuous operation and management

Division	Number of villages (village)	*χ* ^2c^
Major factors affecting project performance		
Understanding and cooperation by residents	125 (40.1)	171.654***
Shared income creation	64 (20.5)	
Support by the government and local government	64 (20.5)	
Manager’s active involvement	26 (8.3)	
Village leader’s outstanding operation ability	24 (7.7)	
Other^a^	9 (2.9)	
Total	312 (100.0)	
Effects of the mountain ecological village project on villages		
Maximization of the forest environmental characteristics of villages	103 (33.0)	82.903***
Maximization of the utilization of forest resources by villages	77 (24.7)	
Achievement of differentiated competitiveness from non-mountain village areas	69 (22.1)	
Increased migration of urban residents	55 (17.6)	
Other	6 (1.9)	
No response	2 (0.6)	
Total	312 (100.0)	
Prerequisites for sustainable operation and management		
Additional support by the government	135 (43.3)	82.000***
Continuous resident education and advertising	50 (16.0)	
Inflow of younger people through the farm/rural-returning induction policy	32 (10.3)	
Other^b^	91 (29.2)	
No response	4 (1.3)	
Total	312.0 (100.0)	

Values in parentheses indicate percentage compositions

^a^Accurate analysis of characteristics and customized investment depending on village, inflow of younger people, and nurturing professional village leaders

^b^Trust and cooperation between village residents, communication between villages, and placement of managers

^c^Chi square goodness of fit test, * p < 0.05, ** p < 0.01, and *** p < 0.001

### Operation and planning of mountain ecological villages

There were 138 operation managers in the 312 villages, who managed various facilities and website operations; 137 villages operated websites. These were mainly used for village advertising, recruiting for mountain village experience activities, and sales of locally produced agricultural and forestry products. The average annual number of advertisements per website was 30 and the average annual number of visitors was 4641 (Table 12).

**Table 12:** Operation managers and website operation

Operation manager (village)		Website (village)		Average annual number of advertisements per website (case)	Average annual number of visitors per website (person)
Present	Absent	Present	Absent		
138	174	137	175	30	4641

When the presence or absence of an event or a long-term development plan for mountain ecological villages was investigated, the ratio of villages with annual events or festivals was as high as 72.8 % of all villages, whereas the ratio of villages with long-term development plans was only 54.2 %. In addition, 78.5 % of all villages participated in village leader education to empower mountain ecological village residents, while people in 61.5 % of all villages attended resident education (Table 13).

**Table 13:** Long-term development plan for village and resident empowerment

Division	Number of villages (village)
Event/festival	
Present	227 (72.8)
Absent	85 (27.2)
Total	312 (100.0)
Long-term development plan for village	
Present	169 (54.2)
Absent	143 (45.8)
Total	312 (100.0)
Participation in village leader education	
Present	245 (78.5)
Absent	67 (21.5)
Total	312 (100.0)
Participation in resident education	
Present	192 (61.5)
Absent	120 (38.5)
Total	312 (100.0)

Values in parentheses indicate percentage compositions

Altogether, 60.3 % of all villages answered “very high” or “high” to the question about the development potential of villages using forest resources, indicating that more than half of villages considered this positive. Satisfaction with job creation after the mountain ecological village development project was “average” for 46.8 % and “low” for 25.3 %, indicating a degree of dissatisfaction. In addition, satisfaction with village income was “average” for 41 % and “low” for 29.5 %. The differences in the categories were all statistically significant (p < 0.001; Table 14).

**Table 14:** Satisfaction with development potential using forest resources and village income

Division	Very high (village)	High (village)	Average (village)	Low (village)	Very low (village)	Average satisfaction (5-point scale)	No response (village)	Total (village)
Development potential using forest resources	47 (15.1)	141 (45.2)	78 (25.0)	37 (11.9)	7 (2.2)	3.6	2 (0.6)	312 (100.0)
*χ* ^2a^	167.290***							
Satisfaction with job creation	12 (3.8)	54 (17.3)	146 (46.8)	79 (25.3)	17 (5.4)	2.9	4 (1.3)	312 (100.0)
*χ* ^2^	193.721***							
Satisfaction with village income	6 (1.9)	77 (24.7)	128 (41.0)	92 (29.5)	7 (2.2)	2.9	2 (0.6)	312 (100.0)
*χ* ^2^	187.774***							

Values in parentheses indicate percentage compositions

^a^Chi square goodness of fit test, * p < 0.05, ** p < 0.01, and *** p < 0.001

Future demand for village income projects using forests mostly comprised recreation and ecotourism using income crop cultivation from forests and wood, including wild greens and wood-cultivated ginseng at 53.5 and 35.9 %, respectively. Desired support from the government for an income promotion project using forests included support for the establishment of forest recreation facilities (25.6 %), national forest lending (20.8 %), provision of new income crops and technical guidance (19.2 %), development of mountain village tourism programs (16.3 %), and continuous support for forest projects (13.8 %). The differences in demand for income projects using forests and desired support items were statistically significant (p < 0.001; Table 15).

**Table 15:** Desired support items from the government for income promotion projects using forests

Division	Number of villages (village)	*χ* ^2c^
Demand for income project using forests		
Development of recreation areas and ecotourism sites using forests	167 (53.5)	478.052***
Cultivation of wood income crops, including wild greens and wood-cultivated ginseng	112 (35.9)	
Lumbering income creation through afforestation	7 (2.2)	
Wood grazing, including Hanwoo and goats	7 (2.2)	
Production and sale of biofuel	4 (1.3)	
Other^a^	13 (4.2)	
No response	2 (0.6)	
Total	312 (100.0)	
Desired support items from the government		
Support for constructing forest recreation facilities	80 (25.6)	53.794***
National forest lending for the income project	65 (20.8)	
Provision for new income crops, technical guidance, and market information	60 (19.2)	
Development and supply of the mountain village tourism program	51 (16.3)	
Continuous support for the surrounding forests by the national forest project	43 (13.8)	
Other^b^	11 (3.5)	
No response	2 (0.6)	
Total	312 (100.0)	

Values in parentheses indicate percentage compositions

^a^Planting fruit trees and seedling production, etc

^b^Workforce support, support for the construction of a distribution center for forest products, continuous management, and operation manager support, etc

^c^Chi square goodness of fit test, * p < 0.05, ** p < 0.01, and *** p < 0.001

## Conclusions

The South Korean government has conducted the mountain ecological village development project since 1995 for the promotion of mountain villages; in addition, it investigated 312 mountain ecological villages for the first time in 2014 in order to ascertain the performance of the project. First, the number of households in the 312 mountain ecological villages increased by 13.5 % at the end of 2013 compared with before the project, while the population barely increased. Indeed, younger age groups, namely, the 20s and 30s groups, decreased by 2.3 %, whereas the 60s or older age groups increased by 2.5 %, indicating aging in mountain villages. The new households were mostly non-farm and non-resident households, who enjoyed rural life during weekends or after retirement rather than treating agriculture as a full-time job. Therefore, these households were not considered to help agroforestry production or forest administration. On the contrary, there was a threefold increase in the inflow of farm/rural-returning and multicultural households compared with before the project, and some of these households were expected to serve as a source of labor supply. However, since most rural-returning people were retirees, their presence might not affect agroforestry production and forest administration. Nevertheless, rural-returning people aged around 60 with stable incomes bases, such as pension incomes, could have positive revitalizing effects on mountain villages, including the expansion of consumption, reduction in depopulation, and exchange of new information and opinions. Furthermore, the increase in the number of farm/village returning and multicultural households was likely attributed to such factors as experience programs and pleasant settlement conditions that mountain ecological villages provide. The influx of the population segment aged 50 years or older was likely because these people wanted to spend the rest of their lives enjoying clean air and quiet and scenic environments. In addition, local governments have been providing training and technical assistance to all intending emigrants for growing agricultural and forest products as well as financial support for housing and agricultural and forest production in order to attract population inflows to mountain ecological villages. In addition, local governments have been providing relevant information on their websites.

On the contrary, living environments, including water supply and sewers were greatly improved, average income per farm increased by 27.8 %, and local product sales and experience tourism income grew remarkably every year. The utilization of forest resources also increased by about 30 % and cultivation of medicinal plants and wood-cultivated ginseng using forests rose greatly. In terms of the performance of the mountain ecological village development project, there was high satisfaction with residential environment improvements and income-related items, and the major factors affecting performance included understanding and cooperation by residents, shared income creation, and government support. However, 45.8 % of the 312 villages had no long-term village development plan, and satisfaction with job creation and village income was average or below average, even though 60.3 % answered “high” to the question of development potential using forest resources. Demand for future income projects using forests were mostly focused on recreation and ecotourism using the forest and the cultivation of wild greens and wood-cultivated ginseng. For these, government support is required, including the construction of recreation facilities, national forest lending, provision of new income crops and technical guidance, development and supply of the mountain village tourism program, and forest project support.

To promote mountain ecological villages, it is first necessary to define the nature of rural- and farm-returning populations clearly. This would enable the determination of their functions and roles in forest management, improving the public function of forests, contribution to economic revitalization through agroforestry production, development of a comfortable national land environment by improving living environments, and contribution to the preservation and development of cultures and heritages of mountain villages. In this way, the above-mentioned factors could be used as important data to establish a promotion policy not only for mountain ecological villages, but also for mountain villages. In addition, it is necessary to understand in detail the urban–rural exchange and experience program carried out to increase farm income and enhance the awareness of the importance of forests.

To ensure the internal substantiality of experience tourism and forest ecotourism in mountain village areas, it is necessary to establish these areas through reviews and discoveries of tangible/intangible cultural assets and traditions in surrounding areas and forests. Furthermore, it is necessary to create a measure to increase farm income by attracting experiences by accommodation type rather than one-day experiences by grouping and regionalizing mountain villages from each mountain village unit. Introducing a measure to explain the values of mountain villages in more detail is also important. In addition, it is necessary to provide the continuous creation of farm income in villages using production infrastructure supported by the mountain ecological village development project with guidance on sales, distribution, and technical matters as well as funding support to raise income.

Development of income sources through the active utilization of forests, government support, and the active renting of national/public-owned forests is necessary to expand wood cultivation, including wild vegetables and medicinal plants, create healing and recreational forests, and build walking and hiking trails. In conclusion, demand for comfortable environments by South Korean people has increased continuously, and mountain villages with plenty of forest resources have positioned themselves as places of recreation and resettlement. Therefore, farm/rural populations returning to mountain villages have increased continuously. In this regard, the mountain villages of South Korea are expected to play a more important role in the future, not only in providing unique natural environments and cultural spaces, but also in supplying clean food and promoting national health. Finally, it is essential for mountain villagers, urban residents, private companies, universities, and non-profit organizations to establish close cooperative relationships in order to achieve the objectives of the mountain ecological village project, which are preservation of forest ecosystems, increase in population inflow, stronger awareness of the importance of mountain villages through urban–rural exchange programs, and increase in the household income of mountain ecological villages using forest resources. Furthermore, various kinds of joint production between cooperatives, farm corporations, social enterprises, and village enterprises should be promoted for the production, processing, and marketing of agricultural and forest products.
